# Characterization of immune checkpoint inhibitor-associated fulminant type 1 diabetes associated with autoantibody status and ethnic origin

**DOI:** 10.3389/fimmu.2022.968798

**Published:** 2022-11-14

**Authors:** Junlin Qiu, Shuoming Luo, Wenfeng Yin, Keyu Guo, Yufei Xiang, Xia Li, Zhenqi Liu, Zhiguang Zhou

**Affiliations:** ^1^ Department of Metabolism and Endocrinology, Key Laboratory of Diabetes Immunology (Central South University), Ministry of Education, National Clinical Research Center for Metabolic Diseases, The Second Xiangya Hospital of Central South University, Changsha, China; ^2^ Division of Endocrinology and Metabolism, Department of Medicine, University of Virginia Health System, Charlottesville, VA, United States

**Keywords:** fulminant type 1 diabetes, immune checkpoint inhibitors, side effects, clinical characteristics, cancer immune checkpoint therapy

## Abstract

**Objective:**

Fulminant type 1 diabetes may uniquely occur as a fatal adverse event during immune checkpoint inhibitor (ICI) therapy. We investigated the clinical and immunological characteristics of ICI-associated fulminant type 1 diabetes (IFD).

**Research design and methods:**

We enrolled 80 patients with IFD (77 cases from the literature), 56 patients with ICI-associated type 1 diabetes (IT1D) (55 cases from the literature), 45 patients with traditional fulminant type 1 diabetes (TFD), and 43 patients with acute-onset type 1 diabetes for comprehensive analysis including islet autoantibodies and subgroup analysis based on ethnic origin.

**Results:**

Patients with IFD accounted for 58.8% (80/136) of patients with ICI-related diabetes. IFD had a more rapid onset than IT1D after ICI therapy (90.5 days *vs.* 120 days, p <0.05). The onset time and number of infusions after ICI therapy initiation were lower in the antibody-positive IFD group than that in the antibody-negative IFD group (both p <0.001). IFD had a more rapid onset and more serious among Caucasians than that among Asians (p <0.01, p <0.05, respectively), and the prevalence of islet autoantibody positivity in the Caucasian IFD were prominently higher than those in the Asian IFD (p <0.05). Onset age and plasma glucose levels were significantly higher in the IFD group than those in the TFD and acute-onset type 1 diabetes groups. HbA1c levels were slightly higher in patients with IFD than those with TFD.

**Conclusions:**

IFD is relatively common in Caucasian population where TFD is very rare or almost absent. IFD occurrence is significantly related to islet autoantibody status and ethnic origin.

## Introduction

Immune checkpoint inhibitors (ICIs), which are the most popular means of tumor immunotherapy, have been increasingly used to treat solid tumors. ICIs have an anticancer effect by removing a negative regulatory signal for T cell activation from the tumor microenvironment. Common ICIs include programmed death 1 (PD-1) inhibitors, programmed death-ligand 1 (PD-L1) inhibitors, and cytotoxic T lymphocyte antigen 4 (CTLA-4) inhibitors. Many studies have confirmed that the application of these ICIs is associated with immune-related adverse events involved in multiple organs and systems. Endocrine dysfunctions are among the common adverse events that have been reported in clinical trials with ICIs, including insulin-dependent diabetes. ICI-associated diabetes is characterized by acute onset of hyperglycemia with insulin deficiency and occurrence following exposure to ICIs. According to the literature, there are two subtypes of insulin-dependent diabetes, namely, ICI-associated type 1 diabetes (IT1D) and ICI-associated fulminant type 1 diabetes (IFD). There has been an increasing number of reports of patients presenting with IT1D and IFD due to the increase of tumor immunotherapy (1–5). If not promptly recognized, IT1D and IFD can be life threatening.

Traditional fulminant type 1 diabetes (TFD) is a rare subtype of type 1 diabetes that differs from acute-onset type 1 diabetes with a distinct entity and unique clinical characteristics, and it may be mediated by multiple factors, including viral infection and pregnancy (6). TFD is characterized by the following symptoms: 1) a remarkably abrupt onset of ketosis or ketoacidosis; 2) a low glycosylated hemoglobin (HbA1c) value despite a high plasma glucose level; and 3) an absence of insulin secretion capacity (7). It remains unclear whether differences exist in clinical phenotypes and immunological characteristics between IFD and TFD. The prevalence and risk of developing IFD following the use of ICIs regimens are also unknown. Furthermore, all clinicians need to be more aware of IFD to prevent deaths due to diabetic ketoacidosis and failure of timely intervention. Therefore, further understanding of the characteristics of IFD patients is needed for improved prognostic and diagnostic application to reduce overall morbidity for this already at-risk population.

Anti-PD-1 agents (nivolumab, pembrolizumab, cemiplimab, sintilimab, and camrelizumab), anti-PD-L1 agents (atezolizumab, avelumab, and durvalumab), and an anti-CTLA-4 monoclonal antibody (ipilimumab) have been reported to cause type 1 diabetes. According to the safety database of a Japanese pharmaceutical company, the incidences of IT1D and IFD were 0.19% and 0.13%, respectively, from July 2014 to August 2017 among 20,600 patients who received nivolumab treatment. Among 3603 patients who received pembrolizumab from December 2016 to August 2017, the incidences of IT1D and IFD were 0.11% and 0.03%, respectively (8). Stamatouli et al. reported that the estimated incidence of type 1 diabetes in a large American medical center was 0.9% (9). In a recent study, Tsang et al. reported that among 538 patients with metastatic melanoma who received anti-PD-1 immunotherapy, 1.9% patients developed type 1 diabetes (10). The World Health Organization (WHO) Safety Report database shows that the number of ICI-related type 1 diabetes patients is increasing (11), which may be related to the increased use of anti-PD-1 and anti-PD-L1 therapies in various cancers. Additionally, combination therapy with CTLA-4 and PD-1 inhibitors may also increase the incidence of IT1D, IFD, and other immune-related adverse events (12). In these cases, the increment of IFD brings challenges to the clinical diagnosis and treatment management of diabetes.

TFD is common in Asians, including Japanese, Koreans, and Chinese, but is rare in Caucasians from the Americas and Europe (13). Patients with IFD have been sporadically reported in China and other Asian countries (14). However, there is increasing number of reported cases of IFD in Caucasians. Thus, it is worth exploring whether there are differences between IFD in Asians and IFD in Caucasians.

Therefore, the present study investigated the clinical and immunological features of IFD by comparing important clinical indexes among four groups of diabetes, namely, IFD, IT1D, TFD, and acute-onset type 1 diabetes. We enrolled 80 patients with IFD (77 cases from the literature), 56 patients with IT1D (55 cases from the literature), 45 patients with TFD, and 43 patients with acute-onset type 1 diabetes for comprehensive analysis, including analysis of islet autoantibodies, and subgroup analysis based on ethnic origin. The present study will provided precise data on the risk of patients with IFD receiving ICI regimens and demonstrated that patients with islet autoantibody positivity or Caucasian ethnic origin are at an increased high risk of IFD.

## Research design and methods

### Patient inclusion and data collection

IFD was defined as fulminant type 1 diabetes induced by exposure to ICIs. TFD was defined as fulminant type 1 diabetes generally associated with viral infection or pregnancy but not associated with ICI. IT1D was defined as type 1 diabetes induced by exposure to ICIs but does not meet the diagnostic criteria for fulminant type 1 diabetes. Acute-onset type 1 diabetes was defined as typical insulin-dependent type 1 diabe with a duration of hyperglycemic symptoms less than 6 months but does not meet the diagnostic criteria for fulminant type 1 diabetes. ICI-associated diabetes included IFD and IT1D. It should be noted that ICI-associated diabetes excluded pre-existing type 2 diabetes or patients with history of diabetes prior to the use of ICI in the present study.

**Figure 1:**
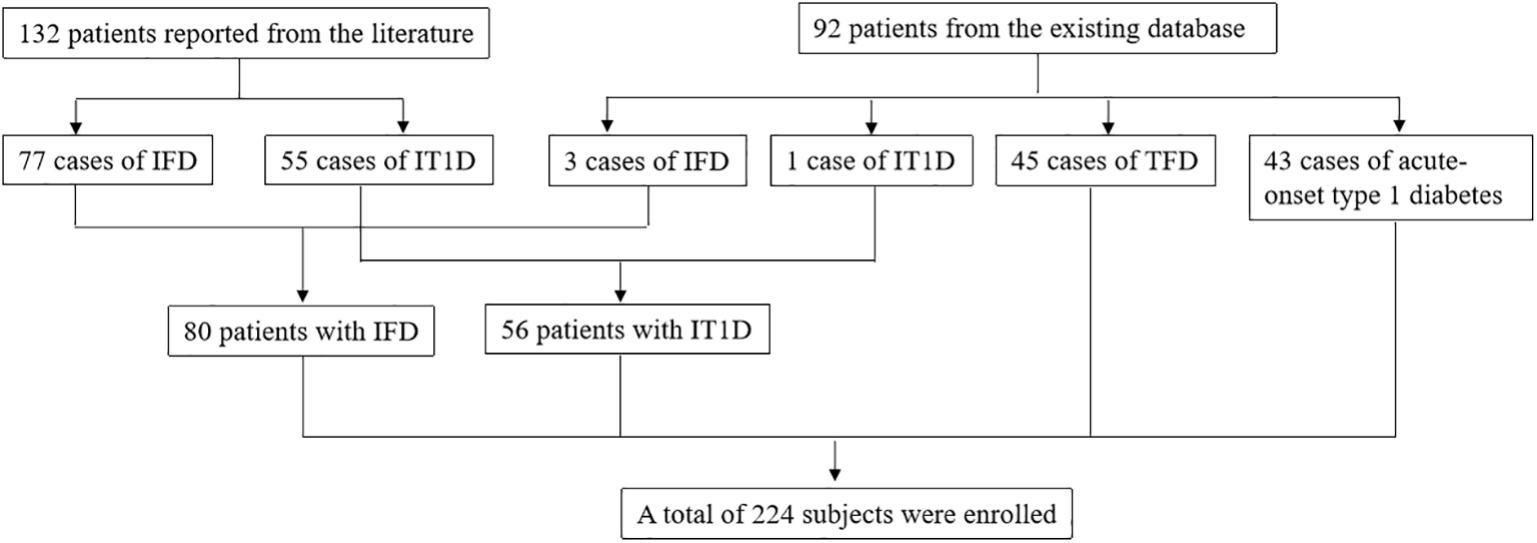
Flow diagram of study participants selection. ICI, Immune checkpoint inhibitor; TFD, Traditional fulminant type 1 diabetes; IFD, ICI-associated fulminant type 1 diabetes; IT1D ICI-associated type 1 diabetes.

A total of 224 subjects were enrolled, including 80 patients with IFD, 56 patients with IT1D, 45 patients with TFD, and 43 patients with acute-onset type 1 diabetes. Among all subjects, 132 patients were reported from the literature, including 77 cases of IFD and 55 cases of IT1D. Three cases of IFD, one case of IT1D, 45 cases of TFD, and 43 cases of acute-onset type 1 diabetes were enrolled from the existing database of our diabetes center (**Figure 1**).

We conducted a systematic search of the literature to identify clinical case reports or articles on the use of ICIs that reported diabetes adverse events. A literature search was used to collect data from patients who developed IFD and IT1D. We gathered data on the clinical characteristics of these cases from literature in online databases, including the CNKI database (Chinese), Wanfang Medical Database (Chinese), and PubMed database. The following keywords were used: “type 1 diabetes mellitus”, “nivolumab”, “pembrolizumab”, “sintilimab”, “toripalimab”, “camrelizumab”, “ipilimumab”, “tremelimumab”, “avelumab”, “durvalumab”, “atezolizumab”, “PD-1”, “PD-L1”, “CTLA-4”, and “immune checkpoint inhibitors”. The CTLA-4 inhibitors included ipilimumab and tremelimumab, and the other inhibitors were PD-1 or PD-L1 inhibitors. The database was searched for articles published on or before December 31, 2021. The search focused on type 1 diabetes related to different ICI regimens in patients with advanced solid tumors. The exclusion criterion was duplication of data. Case reports/series of individuals previously diagnosed with type 2 diabetes prior to the start of ICI therapy and case reports of individuals without confirmed diagnosis of diabetes type were also excluded. Two researchers read and evaluated the literature independently. A third individual was consulted to reach a consensus in cases when both researchers differed on the inclusion or exclusion decision. Ultimately, 99 articles and 132 patients, including 77 patients with IFD and 55 patients with IT1D, between January 2014 and December 2021 were enrolled.

Fulminant type 1 diabetes met the following diagnostic criteria of the Committee of the Japan Diabetes Society in 2012: 1) diabetic ketosis or ketoacidosis occurred soon after the onset of hyperglycemic symptoms; 2) patient presented with plasma glucose ≥16.0 mmol/L and HbA1c <8.7% at the first visit; and 3) patient had urinary C-peptide excretion <10 µg/day, fasting serum C-peptide level <0.10 nmol/L, or postprandial serum C-peptide <0.17 nmol/L at onset (15).

Data on the following parameters were recorded for each patient: demographic data (including sex, onset age, and body mass index (BMI)), tumor types, past history, family history, date of diabetes onset, hyperglycemic symptoms, number of ICI therapy infusions, and types of ICI therapy. The following laboratory data were recorded at onset: plasma glucose, electrolytes, blood gas analysis results, HbA1c, type 1 diabetes-associated autoantibody status, and human leukocyte antigen (HLA) class II alleles or genotypes if available. HLA typing was performed in a subset of the published IFD or IT1D cases. We determined whether this allele or genotype belonged to the susceptibility of spontaneous type 1 diabetes according to the corresponding literature as a reference. For example, DR3-DQ2 and DR4-DQ8 confer increased risk for Caucasian population, while DR4-DQ4 and DR9-DQ9 confer high risk for Asian populations. The onset date was defined as the day of diagnosis and commencement of treatment for diabetes. The unit of C-peptide was uniformly converted into ng/ml, and the unit of blood glucose was uniformly converted into mg/dl. The detection methods of islet autoantibodies, HbA1c, and HLA typing from most cases reported in the literature were unavailable.

### Statistical analysis

All statistical analyses were performed using SPSS version 19.0 (IBM Corporation, Chicago, IL, USA). Continuous variables that were normally distributed are presented as means ± standard deviations (SDs), and continuous variables that were not normally distributed are described as the median and interquartile range (IQR). Differences between groups were analyzed using independent sample t tests, rank sum tests, or variance analysis as appropriate. The chi-squared test was used for correlation analysis of categorical variables. Other clinical variables of interest were evaluated descriptively. According to the comparison results of IFD and IT1D, a logistic regression model was used to incorporate antibody, race, and HLA susceptibility alleles to analyze the predictors of IFD after ICI treatment. For all computational analyses, p < 0.05 was considered statistically significant.

## Results

### Background of patients with IFD

**Figure 2:**
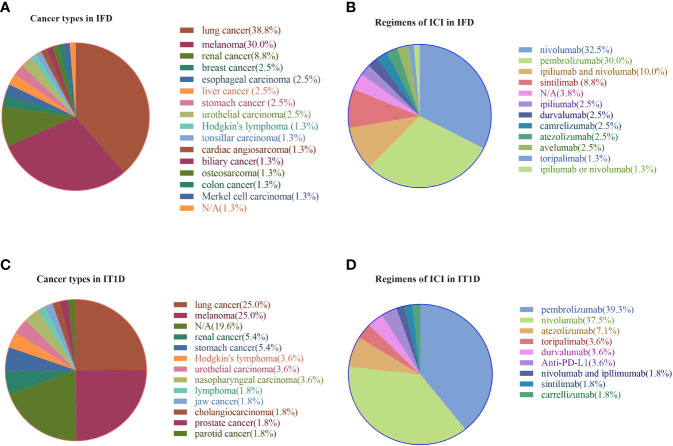
Profile of primary cancer types and ICI regimens in patients with IFD and IT1D. **(A)** Cancer types in IFD, **(B)** Regimens of ICI in IFD, **(C)** Cancer types in IT1D, **(D)** Regimens of ICI in IT1D. IFD, ICI-associated fulminant type 1 diabetes; IT1D, ICI-associated type 1 diabetes; N/A, Not Available.

Patients with IFD accounted for 58.8%(80/136) of patients with ICI-associated diabetes. The onset age of patients with IFD was 60.7 ± 12.6 years, and the BMI was 22.0±5.1 kg/m^2^. Both IFD and IT1D mainly involved with anti-PD-1 and anti-PD-L1 agents was associated with the treatment of various malignancies and various ICI drugs. Regarding the profile of primary cancers (**Figure 2A**), lung cancer, melanoma, and renal cancer accounted for 38.8%, 30.0%, and 8.8%, respectively, while the remaining 22.2% was attributed to other cancer types for IFD. Similarly, lung cancer (25.0%) and melanoma (25.0%) accounted for the highest proportion for IT1D (**Figure 2C**). As shown in **Figures 2B**, **D**, nivolumab (32.5%) and pembrolizumab (30.0%) were the most common tumor immunotherapy regimens in IFD patients. Similarly, pembrolizumab (39.3%) and nivolumab (37.5%) were the most common ICI regimens for IT1D. A summary of the case reports for IFD and IT1D is shown in **Supplemental Tables 1**, **2**, respectively.

IFD was reported in 17 countries on a global scale, including UK, Greece, Belgium, France, Australia, Portugal, Austria, Canada, Italy, Norway, USA, Brazil, Ireland, Spain, Japan, Korea, and China. IFD patients were diagnosed with a median of 90.5 days (IQR, 36.5-150 days), and they a median of five infusions (IQR, 2.3-8 infusions) after initiation of ICI therapy. The duration of symptoms at onset in patients with IFD was 5 days (IQR, 3-8 days). All patients had marked hyperglycemia (638.77±244.29 mg/dl), low C-peptide levels [0.06 (0.01-0.16) ng/ml], and low HbA1c levels (7.36±0.75). All patients with IFD exhibited abrupt onset of ketosis or ketoacidosis (arterial pH: 7.18 ± 0.16, HCO_3_^–^: 12.07±6.94 mmol/L), and 80.3% of patients presented with diabetic ketoacidosis. All patients with IFD were insulin dependent.

### Islet autoantibodies and HLA typing

Sera collected at diabetes onset were tested for type 1 diabetes-related autoantibodies. Islet autoantibodies testing results were available for 77 of 80 patients with IFD, among which the percentage of patients who were positive for at least one autoantibody was 26.0% (20/77) (**Supplemental Table 1**). The autoantibody with the highest positive rate was glutamic acid decarboxylase antibody (GADA), which was found in 21.1% (15/71) of patients, followed by protein tyrosine phosphate antibody (IA-2A) (17.5%, 7/40), insulin autoantibody (IAA) (8.3%, 3/36), islet cell autoantibody (ICA) (0.04%, 1/25), and zinc transporter 8 autoantibody (ZnT8A) (6.3%, 1/16).

In total, 36 patients with IFD underwent HLA typing, and 58.3% of these patients had the high-risk HLA genotype for type 1 diabetes. As shown in **Table 1**, the proportions of type 1 diabetes HLA susceptibility alleles were not statistically different between IFD and IT1D. The proportion of type 1 diabetes HLA susceptibility alleles was higher in the antibody-positive IFD group compared to the antibody-negative IFD group, but there was no statistically significant difference when comparing the different ethnic IFD subgroups.

**Table 1:** The clinical and biological characteristics of different ICI-associated diabetes.

	IFD	IT1D	P
N	80	56	
Onset age (years)	60.7 ± 12.6	63.4 ± 13.0	NS
Sex (male %)	55.7	76.1	0.023
BMI (kg/m^2^)	22.0±5.1	28.1 ± 7.4	0.003
Family history of diabetes (%)	15	23.5	NS
Time from initiation of therapy to onset of diabetes (days)	90.5 (36.5, 150)	120 (63, 270)	0.017
Number of courses before diabetes onset	5.0 (2.3, 8.0)	5 (3.0, 10.5)	NS
Diabetic ketoacidosis (%)	80.3	58.0	0.007
Plasma glucose (mg/dl)	638.77 ± 244.29	585.34 ± 235.71	NS
HbA1c (%)	7.36 ± 0.75	9.05 ± 1.32	0.000
Arterial PH	7.18 ± 0.16	7.22 ± 0.16	NS
HCO_3_^-^(mmol/l)	12.07± 6.94	15.27 ± 7.35	NS
Serum C-peptide (ng/ml)	0.06 (0.01, 0.16)	0.46 (0.20, 0.99)	0.000
Islet autoantibodies (%)	26.0	46.0	0.017
^&^Proportion of HLA susceptibility alleles for type 1 diabetes (%)	58.3 (21/36)	75(12/16)	NS

Values are expressed as the mean ± standard deviation or median (first quartile–third quartile).

IFD, Immune checkpoint inhibitor associated fulminant type 1 diabetes; IT1D, Immune checkpoint inhibitor associated type 1 diabetes but not fulminant type 1 diabetes; NS, no significance.

^&^ HLA typing was performed in a subset of the published IFD or IT1D cases. We determine whether this allele or genotype belongs to the susceptibility of spontaneous type 1 diabetes on their own according to the corresponding literature as a reference. For example, DR3-DQ2 and DR4-DQ8 confer increased risk for Caucasian population, while DR4-DQ4 and DR9-DQ9 confer high risk for Asian populations.

### Comparison of clinical characteristics between IFD and IT1D

To further understand whether the clinical features of IFD are specific, we compared the clinical and biological characteristics of IFD and IT1D. Compared to IT1D, the percentage of men was lower in IFD (p < 0.05), whereas the percentage of patients with diabetic ketoacidosis was significantly higher in IFD (p < 0.05) (**Table 1**). The time from initiation of ICI therapy to onset of diabetes in patients with IFD was significantly less than that in patients with IT1D (90.5 days vs. 120 days, p < 0.05). However, no statistically difference was observed for the median number of ICI infusions before diabetes onset (5 infusions vs. 5 infusions). Patients with IFD displayed a lower BMI, lower HbA1c levels, and lower serum C-peptide levels than those with IT1D (all p < 0.01). Compared to IT1D, the prevalence of islet autoantibodies in IFD also had a significant decreasing trend (p = 0.017). There was no significant difference between IFD and IT1D with respect to onset age, family history of diabetes, plasma glucose, arterial PH, and HCO3^-^ levels.

According to the comparison results, there was a difference in the positive ratio of islet autoantibodies between the IFD and IT1D groups. In the present study, correlation analysis showed that autoantibody status in patients with IFD was associated with ethnicity (**Table 2**). Therefore, a logistic regression model, including three variables (antibody, race, and susceptibility genotype) was used to evaluate the predictors of IFD after ICI treatment (**Supplemental Table 3**). Unexpectedly, we were unable to identify any interactions or predictive risk factors for IFD and IT1D (all p > 0.05).

**Table 2:** Correlation analysis of autoantibody and ethnic origin in IFD.

	Ethnic origin		Total	c^2^	r	P value
	Asian	Caucasian				
Autoantibody -positive	4	16	20	11.19	0.380	0.001
Autoantibody -negative	38	19	57			

IFD, Immune checkpoint inhibitor associated fulminant type 1 diabetes.

### Clinical characteristics of different subgroups of IFD

Subgroup analysis was performed for patients with IFD according to their autoantibody status and ethnic origin. As shown in **Table 3**, the median number of infusions and time from initiation of ICI therapy to onset of diabetes in the antibody-positive IFD group were significantly lower than those in the antibody-negative IFD group (2 infusions vs. 6 infusions, p < 0.001; 28.5 days vs. 114 days, p < 0.001).

**Table 3:** The clinical and biological characteristics of IFD in different autoantibody status.

	Autoantibody-positive IFD	Autoantibody-negative IFD	P
N	20	57	
Age (years)	57.4 ± 17.6	61.2 ± 10.3	NS
Sex (male %)	36.8	63.2	0.039
BMI (kg/m^2^)	24.7 ± 7.3	20.8 ± 3.3	NS
Time from initiation of therapy to onset of diabetes (days)	28.5 (20.3, 40.3)	114 (71, 168)	0.000
Number of courses before diabetes onset	2 (1, 3.5)	6 (4, 9)	0.000
Diabetic ketoacidosis (%)	89.5	75.9	NS
Plasma glucose (mg/dl)	662.51 ± 262.49	619.83 ± 239.74	NS
HbA1c (%)	7.14 ± 0.77	7.39 ± 0.71	NS
Arterial PH	7.19 ± 0.16	7.18 ± 0.16	NS
HCO_3_^-^	11.33 ± 5.06	12.63 ± 7.52	NS
Serum C-peptide (ng/ml)	0.1 (0.02, 0.1)	0.05 (0.1, 0.17)	NS
Proportion of HLA susceptibility alleles for type 1 diabetes (%)	88.9 (8/9)	44.0 (11/25)	0.01

Values are expressed as the mean ± standard deviation or median (first quartile–third quartile).

IFD, Immune checkpoint inhibitor associated fulminant type 1 diabetes; NS, no significance.

In the present study, 45.0% (36/80) of the patients were Caucasians from Belgium, Italy, Greece, and 13 other countries, whereas 55.0% (44/80) of the patients were Asians from the UK (South-East Asian origin), Japan, Korea, and China (**Supplemental Table 1**). As shown in **Table 4**, Caucasian patients with IFD had a lower median number of infusions and a more rapid onset than Asian patients with IFD (3 infusions vs. 6 infusions, p < 0.05; 40 days vs. 110 days, p < 0.01), and the proportions of diabetic ketoacidosis and positive rate of autoantibodies in Caucasian patients with IFD were significantly higher than those in Asian patients with IFD (91.2% vs. 71.4%, p < 0.05; 45.7% vs. 9.5%, p < 0.01). Correlation analysis showed that autoantibody status in patients with IFD may be associated with ethnicity of patients with IFD (**Table 2**).

**Table 4:** The clinical and biological characteristics of IFD in different ethnic origin.

	Asian origin	Caucasian origin	P
N	44	36	
Age (years)	62.9 ± 11.2	58.0 ±13.9	NS
Sex (male %)	54.5	57.1	NS
BMI (kg/m2)	20.2 ± 3.5	24.3 ± 6.0	NS
Time from initiation of therapy to onset of diabetes (days)	110 (72, 171)	40 (28, 128)	0.007
Number of courses before diabetes onset	6 (4.5, 8)	3 (2, 9)	0.033
Diabetic ketoacidosis (%)	71.4	91.2	0.029
Plasma glucose (mg/dl)	629.23 ± 260.75	650.42 ± 225.65	NS
HbA1c (%)	7.37 ± 0.75	7.36 ± 0.76	NS
Arterial PH	7.19 ± 0.17	7.16 ± 0.16	NS
HCO_3_^-^	13.13 ± 7.36	10.95 ± 6.45	NS
Serum C-peptide (ng/ml)	0.03 (0.01, 0.1)	0.10 (0.02, 0.19)	NS
Islet autoantibodies (%)	9.5 (4/42)	45.7 (16/35)	0.001
Proportion of HLA susceptibility alleles for type 1 diabetes (%)	68.4 (13/19)	47.1 (8/17)	NS

Values are expressed as the mean ± standard deviation or median (first quartile–third quartile).

IFD, Immune checkpoint inhibitor associated fulminant type 1 diabetes; NS, no significance.

### Comparison of clinical characteristics among IFD, TFD, and acute-onset type 1 diabetes

To eliminate the influence of racial differences, we selected 20 patients with IFD (17 cases from the literature and 3 case from our existing database), 45 patients with TFD, and 43 patients with acute-onset type 1 diabetes for comparison of clinical characteristics, and all of these patients were Chinese. As shown in **Table 5**, the onset age, plasma glucose levels, and proportion of patients with diabetic ketoacidosis at onset in the IFD group were significantly higher than those in the acute-onset type 1 diabetes group (all p<0.001). The duration of symptoms, fasting C-peptide levels, mean HbA1c levels, and prevalence of positive autoantibody in the IFD groups were significantly lower than those in the acute-onset type 1 diabetes group (all p<0.001). The onset age, plasma glucose levels, and HbA1c levels in patients with IFD were significantly higher than those in patients with TFD (58.0 ± 9.3 years vs. 31.0 ± 13.9 years, 680.22 ± 283.83 mg/dl vs. 557.90 ± 198.73 mg/dl, and 7.68% ± 0.60% vs. 6.81% ± 0.86%, respectively, all p<0.001).

**Table 5:** Comparison of the clinical and biological characteristics among IFD, TFD, and acute-onset T1D in Chinese patients.

	TFD	IFD	acute-onset T1D
N	45	20	43
Onset Age, years	31.0 ± 13.9^*^	58.0 ± 9.3^*#^	24.2 ± 17.0
BMI (kg/m2)	22.0 ± 3.7	21.2 ± 3.2	19.4 ± 3.7
Male (%)	57.8	75.0	60.5
Duration of symptoms (days)	3 (2, 5)^*^	6 (3, 7)^*^	30 (10, 34)
Diabetic ketoacidosis (%)	77.8^*^	85.0^*^	48.8
Plasma glucose (mg/dl)	557.90 ± 198.73^*^	680.22 ± 283.83^*#^	449.33 ± 118.53
Arterial PH	7.14 ± 0.28	7.17 ± 0.13	7.25 ± 0.15
HCO_3_^-^	10.14 ± 5.82	11.77 ± 4.09	12.98 ± 6.95
Serum C-peptide (ng/ml)	0.05 (0.03, 0.15)^*^	0.01 (0.01, 0.05)^*^	0.32 (0.14, 0.54)
HbA1c (%)	6.81 ± 0.86^*^	7.68 ± 0.60^*#^	12.41 ± 2.59
Islet autoantibodies (%)	14.0 (6/43)^*^	10.5 (2/19)^*^	72.1 (31/43)

Values are expressed as the mean ± standard deviation or median (first quartile–third quartile).

T1D, type 1 diabetes.

TFD, Traditional fulminant type 1 diabetes.

IFD, Immune checkpoint inhibitor associated fulminant type 1 diabetes.

^*^P <0.05, vs. acute-onset T1D; ^#^P<0.05, vs. TFD.

## Discussion

ICIs, especially PD-1 inhibitors, can cause type 1 diabetes as an immune adverse event, which is usually accompanied with severe complications, such as diabetic ketoacidosis. The present study comprised the largest sample size of IFD patients to date. The present results indicated that IFD was not uncommon in patients receiving ICI treatment, especially among Caucasians. Nivolumab and pembrolizumab were the most common ICIs leading to diabetes, and the most common tumor types were melanoma and lung cancer. The median HbA1c was low, suggesting abrupt onset of diabetes. The proportion of IFD patients with positive autoantibodies was 26.0%, and GADA was the most prevalent diabetes-associated autoantibody. Autoantibody-positive IFD patients showed faster onset due to a lower median number of infusions and time from initiation of ICI therapy to onset of diabetes. Interestingly, Caucasians with IFD had a more rapid onset and more serious disease than Asians with IFD. At the same time, the antibody-positive rate of the Caucasian population was higher than that of the Asian population. However, it remains unknown whether the high proportion of autoantibodies in the Caucasian population causes different conditions and disease progression from those in the Asian population, thereby additional studies are warranted.

The increased number of reports of ICI-related diabetes, which are mainly related to the use of PD-1 or PDL-1 inhibitors, has provoked widespread concern. The exact mechanisms of these cases of acute insulin-dependent diabetes are currently unknown. The PD-1/PDL-1 axis affects islet autoimmunity through different mechanisms involving innate and adaptive immune cells, and these affects occur in draining lymph nodes and pancreatic tissue (16). The rarity of these secondary diabetes events makes them challenging to characterize. However, if severe hyperglycemia is not detected and treated in time, the patient is likely to die from diabetic ketoacidosis rather than the malignant tumor. Therefore, it is necessary to summarize the IFD patients reported all over the world and conduct comprehensive analyses to identify early prediction risk factors.

Recent studies have found that islet autoantibodies, especially GADA, which is considered the main autoantibody in patients with type 1 diabetes, are related to IFD. However, GADA negativity does not indicate that other islet autoantibodies are negative, suggesting that other antibodies may be positive. In the present study, the prevalence of islet autoantibodies was 26.0% and 46.0% in IFD and IT1D, respectively. Similar to this study, Clotman et al. found that 56% of patients with ICI related diabetes are positive for islet autoantibodies, including GADA (17). De Filette et al. reported that at least one autoantibody is positive in 53% of ICI related diabetes patients with GADA having the highest positive rate (51%) (18). It has been reported that an antibody-positive ICI related diabetes group has a more rapid onset and higher incidence of diabetic ketoacidosis compared to an antibody-negative ICI related diabetes group (19). The median time from ICI treatment to the diagnosis of type 1 diabetes is 5 weeks for GADA-positive cases and 9 weeks for GADA-negative cases (17). GADA-positive patients use ICIs for a median of 3.1 cycles, while GADA-negative patients use ICIs for 5.9 cycles (18). In line with this, the present study demonstrated that patients in the autoantibody-positive IFD group had a significantly lower number of median infusions and time from initiation of ICI therapy to onset of diabetes compared to patients in the autoantibody-negative IFD group. These findings provide evidence supporting the importance of detection of islet autoantibodies for patients before and after using ICI therapy.

Studies have determined that islet autoantibodies are not directly involved in disease pathogenesis (20) but that they precede and predict the development of clinical diabetes (21). In some patients, islet autoantibodies may be present prior to type 1 diabetes (22, 23), whereas in other patients who develop type 1 diabetes, seroconversion may occur after the initiation of ICI therapy (24, 25). Some researchers have suggested that baseline autoimmune antibodies may not be particularly useful as biomarkers to predict individual susceptibility to ICI related diabetes (26), whereas others have suggested that the presence of islet autoantibodies prior to treatment may predispose patients to the development of autoimmune diabetes (23). In our opinion, the absence of diabetes-related autoantibodies cannot rule out the occurrence of IFD, but in such cases, the onset will be slower than that of cases with positive autoantibodies. Autoantibodies are usually considered a biomarker of islet cell destruction. A previous prospective study with a 3-year follow-up has shown that islet autoantibodies may accelerate the decline in β cell function (27). Therefore, we suspect that ICIs inhibit immune tolerance, leading to T cell activation and loss of immune tolerance to B cells, producing islet autoantibodies. However, B cells are not necessarily involved in all patients, that is, not all patients have islet autoantibodies. If B cells are involved, the onset of type 1 diabetes will occur sooner. In the present study, most autoantibodies were measured at the onset of diabetes, and only one patient had antibody data before the onset of diabetes. Interestingly, a frozen blood sample obtained prior to treatment with nivolumab has been shown to be positive for islet autoantibodies despite no prior history of diabetes and no family history of diabetes (23). ICI may have simply accelerated a pre-existing autoimmune process that ultimately led to the development of type 1 diabetes in this patient. If the autoantibody is found to be positive at first, timely intervention will reduce the chance of ketoacidosis at the onset of the disease. In addition, the presence of both GADA and IA-2A in the first degree relatives of patients with type 1 diabetes has been shown to confer a 61% risk of developing type 1 diabetes in 10 years (28). However, the incidence of type 1 diabetes is low in Asian populations. The prevalence of islet-specific autoantibodies has been reported to be lower in Asians compared to Caucasians (29). In the present study, the prevalence of autoantibodies in Caucasian patients with IFD was also higher than that in Asian patients with IFD. Thus, if islet autoantibodies can be a predictor of IFD, it may not work as well in Asian populations as in Caucasian populations.

The HLA class II gene is the most important susceptibility gene for type 1 diabetes. The most common allele in patients with IT1D is HLA-DR4 (9, 18). A previous study has shown that the DRB1*0405-DQB1*0401 and DRB1*0901-DQB1*0303 haplotypes contribute to the susceptibility to fulminant type 1 diabetes (30). Unfortunately, no available detailed haplotypes could be analyzed in most of the IFD cases in the present study. On a different basis, we determined that the proportion pof type 1 diabetes HLA susceptibility alleles was high regardless if the patient had IFD or IT1D, which suggested that type 1 diabetes HLA susceptibility alleles may be predictors of IFD. However, the effect of HLA susceptibility genes on IFD remains unknown. In the future, large sample size case-control studies are needed to evaluate the correlation between HLA susceptibility gene and IFD.

TFD is a rare subtype of type 1 diabetes that is especially prevalent in east Asians and rare in western Caucasians. In the present study, we found an interesting phenomenon that IFD was not infrequent in the Americas and Europe with a proportion of 45.0% in the present dataset. According to previous reports, the rate of islet autoantibody positivity may be relatively lower among Japanese individuals with ICI related diabetes than that among Caucasians (4.76% vs. 56%) (8). In the present study, the rate of autoantibody positivity in Asian IFD patients was also lower than that in Caucasian IFD patients. Compared to Asians with IFD, Caucasians with IFD had a more rapid onset and a higher proportion of diabetic ketoacidosis, which may be attributed to their higher rate of autoantibody positivity. In the present study, the correlation analysis supported this conjecture that autoantibody status in patients with IFD is associated with ethnic origin. More prospective studies are needed in the future for confirmation of these results.

The global increase in ICI use across cancer types highlights the importance of early monitoring and identification of IFD as well as increasing awareness for clinicians. The Japanese Diabetes Association recommends that patients receiving ICI therapy should have their blood glucose levels checked at each visit (every 2-3 weeks). The American Society of Clinical Oncology recommends that blood glucose levels should be measured during each course of treatment for 12 weeks during the induction period and every 3-6 weeks after ICI therapy (31). The present findings indicated that IFD can occur at various time points during the use of ICIs. According to the present study, the currently advocated responses may be insufficient to detect IFD early and reduce its severity at onset because IFD often presents with diabetic ketoacidosis within a week of diabetes symptoms. For early diagnosis and treatment of IFD, we propose the following procedures: 1) blood glucose should be monitored every week during ICI treatment; 2) diabetes-associated autoantibodies should be tested before or during treatment with ICIs, and if the autoantibodies are positive, it is necessary to pay close attention to the symptoms of hyperglycemia and strengthen the frequency of blood glucose monitoring to protect against the occurrence of diabetic ketoacidosis; 3) before initiation of ICI treatment, the patient should be informed of the rare possibility of insulin-dependent diabetes and the corresponding countermeasures; and 4) when conditions permit, the detection of type 1 diabetes susceptibility gene should be considered.

The present study had important strengths. Although obtaining IFD cases is difficult because IFD is rare, the novel design of the present study allowed collection of detailed case information by searching the literature to obtain a considerable sample size for analysis and research. We focused on fulminant type 1 diabetes as an adverse event of ICI therapy and found several interesting results. These results highlighted that the occurrence of IFD is related to autoantibody status and ethnic differences as IFD patients with positive islet autoantibodies or patients with Caucasian ethnicity have a more rapid onset.

The present study had several limitations. Most cases were obtained from existing literature, which led to incomplete clinical data, such as lack of pancreatic enzymes and daily dose of insulin. The consistency of the detection methods of some important parameters, such as islet autoantibodies and HbA1c, was not guaranteed, resulting in potential confounding factors in statistical analysis. There was also a lack of sufficient comparable HLA genotype information. Clearly, if we had conducted HLA typing in the form of case-control study, it would have allowed greater insight into the IFD risk associations. In addition, the analysis in the present study was cross-sectional, allowing the predictive potential of autoantibodies to be inferred from the clinical state only at the time of observation. Additional longitudinal cohort studies are required to demonstrate the clinical usefulness of autoantibodies. Finally, larger studies will improve the IFD risk prediction associated with autoantibodies or susceptibility genes.

In summary, IFD is relatively common in the Caucasian population, in which TFD is rare or almost absent. The present data suggested that IFD occurrence may be significantly related to autoantibody status and ethnic differences. Patients with positive islet autoantibodies or Caucasians have a more rapid and more serious onset. Prospective studies are needed to identify more effective risk prediction methods for developing ICI-induced diabetes. Due to the rapid onset of IFD, all acutely unwell patients on ICI should have their blood glucose checked and a full work-up for diabetes ketoacidosis if necessary.

## Data availability statement

The original contributions presented in the study are included in the article/**Supplementary Material**. Further inquiries can be directed to the corresponding authors.

## Ethics statement

All procedures performed in studies involving human participants were in accordance with the ethical standards of the institutional and/or national research committee and with the 1964 Helsinki Declaration and its later amendments or comparable ethical standards. The patients/participants provided their written informed consent to participate in this study. Our study (2022 Scientific Research Ethics Review No. 31) was approved by the ethics review board of National Clinical Research Center for Metabolic Diseases at the Second Xiangya Hospital of Central South University.

## Author contributions

JQ collected and researched data and wrote manuscript, SL researched data, edited the manuscript, and contributed to discussion, ZZ reviewed manuscript and contributed to discussion, WY and KG collected data and contributed to discussion, YX, XL, and ZL reviewed the manuscript and contributed discussion. All authors approved the final manuscript. SL and ZZ are the guarantor of this work and, as such, had full access to all the data in the study and take responsibility for the integrity of the data and the accuracy of the data analysis. All authors contributed to the article and approved the submitted version.

## Funding

This study was supported by key projects supported by the Hunan Health Commission (Grant No. 202103060904), Hunan Province Natural Science Funds (Grant No. 2020JJ2053), and independent exploration and innovation projects for postgraduate of Central South University (Grant No. 2021zzts1052).

## Conflict of interest

The authors declare that the research was conducted in the absence of any commercial or financial relationships that could be construed as a potential conflict of interest.

## Publisher’s note

All claims expressed in this article are solely those of the authors and do not necessarily represent those of their affiliated organizations, or those of the publisher, the editors and the reviewers. Any product that may be evaluated in this article, or claim that may be made by its manufacturer, is not guaranteed or endorsed by the publisher.

## Abbreviations

GADA, Glutamic acid decarboxylase autoantibodies; IA-2A, Protein tyrosine phosphatase autoantibodies; ICA, Islet cell autoantibodies; ICI, Immune checkpoint inhibitor; IFD ICI-associated fulminant type 1 diabetes; IT1D ICI-associated type 1 diabetes; TFD, Traditional fulminant type 1 diabetes; ZnT8A, Zinc transporter 8 autoantibodies.
