# Supplementary material for: Characterization of immune checkpoint inhibitor-associated fulminant type 1 diabetes associated with autoantibody status and ethnic origin
Source: Front Immunol. 2022 Nov 14;13:968798. doi: 10.3389/fimmu.2022.968798 (PMC9702060; doi:10.3389/fimmu.2022.968798)
Supplement: Supplementary file 1 [file Table_1.doc]

**Supplemental tables**

**Supplemental table 1** Summary of Case Reports of Immune checkpoint inhibitor induced fulminant type 1 diabetes (IFD)

| case | Literature | Onset Age/sex | country | Primary  diagnosis | ICI drugs | Doses | DKA | Diagnosis | | Islet antibodies | HLA susceptibility genes for type 1 diabetes |
| --- | --- | --- | --- | --- | --- | --- | --- | --- | --- | --- | --- |
|  |  |  |  |  |  |  |  | C-peptide  (ng/ml) | HbA1c  (%) |  |  |
| 1 | Hughes, et al.2015(1) | 55/F | USA | melanoma | nivolumab | N/A | + | 0.1 | 6.9 | ICA (-), IAA (-), GADA (-) | (+) |
| 2 | Hughes, et al.2015(1) | 83/F | USA | lung cancer | nivolumab | N/A | + | 0.1 | 7.7 | GADA (+), ICA (-), IAA (-) | (+) |
| 3 | Gaudy, et al.2015(2) | 44/F | France | melanoma | pembrolizumab | 2 | + | 0.01 | 6.8 | GADA (-), IA-2A (-) | N/A |
| 4 | Miyoshi, et al.2016(3) | 66/F | Japan | melanoma | nivolumab | 6 | + | 0.23 | 7.3 | GADA (-), IA-2A (-), ZnT8 (-) | (-) |
| 5 | Aleksova, et al.2016(4) | 60/M | Australia | melanoma | pembrolizumab | 2 | + | 0.17 | 7.1 | GADA (-), IA-2A (-) | N/A |
| 6 | Usui, et al.2017(5) | 62/F | Japan | lung cancer | nivolumab | 4 | N/A | urinary C-peptide2.6ug/d | 6.5 | GADA (-) | (+) |
| 7 | Usui, et al.2017(5) | 31/M | Japan | lung cancer | nivolumab | 1 | + | 0.03 | 6.4 | GADA (+) | (+) |
| 8 | Munakata, et al.2017(6) | 72/M | Japan | Hodgkin’s lymphoma | nivolumab | 6 | - | urinary C-peptide5.0ug/d | 7.3 | GADA(-), ICA (-), ZnT8 (-) | (+) |
| 9 | Kong, et al.2016(7) | 68/M | Korea | lung cancer | pembrolizumab | 7 | + | 0.09 | 7.9 | GADA(-),ICA(-), IAA (-) | (+) |
| 10 | Sicheng Li, 2018(8) | 67/M | China | lung cancer | pembrolizumab | 3 | + | 0.1 | 8.0 | ICA (-), IAA (-), GADA (-) | N/A |
| 11 | Araújo, et al.2017(9) | 73/F | Portugal | lung cancer | nivolumab | 2 | + | 0.06 | 7.2 | GADA (+),ICA (-) | (+) |
| 12 | Tsiogka, et al.2017(10) | 64/M | Austria | melanoma | ipilimumab | 3 | + | 0.01 | 6.1 | ICA (-), IAA (-), GADA (-), IA-2A (-) | (-) |
| 13 | Takahashi, et al.2018(11) | 74/M | Japan | melanoma | nivolumab | 6 | + | urinary C-peptide<0.6 ug/d | 8.0 | IAA (-), GADA (-) | N/A |
| 14 | Teló, et al.2017(12) | 51/M | Brazil | renal carcinoma | ipilimumab and nivolumab | 2 | + | 0.01 | 7.2 | GADA (-), IA-2A (-) | N/A |
| 15 | Ishikawa, et al.2017(13) | 54/F | Japan | melanoma | nivolumab | 16 | N/A | 0.1 | 7.0 | GADA (-), IA-2A (-) | (+) |
| 16 | Mizab Mellah C, et al.2017(14) | 58/M | Spain | melanoma | pembrolizumab | 4 | + | 0.02 | 7.4 | GADA (-), IA-2A (-) | N/A |
| 17 | Godwin, et al.2017(15) | 34/F | USA | lung cancer | nivolumab | 2 | + | 0.1 | 7.1 | GADA (+), IAA (-),  IA-2A (+), ZnT8 (+) | (-) |
| 18 | Chae ,et al.2017(16) | 76/M | USA | lung cancer | pembrolizumab | 1 | N/A | 0.1 | 5.8 | GADA (+), IA-2A (+), IAA (-), ICA (-) | N/A |
| 19 | Leonardi, et al.2017(17) | 66/M | USA | lung cancer | pembrolizumab | 3 | N/A | 0.1 | 7.6 | GADA (+), IAA (-), ICA(-) | N/A |
| 20 | Sakurai, et al.2018(18) | 68/F | Japan | renal carcinoma | nivolumab | 1 | - | 0.24 | 6.9 | IAA (-), GADA (-), IA-2A (-), ZnT8 (-) | (+) |
| 21 | Shiba, et al.2018(19) | 80/F | Japan | melanoma | ipilimumab | 1 | - | 0.01 | 7.7 | IAA(+),GADA(-),IA-2A (-) | (+) |
| 22 | Sakai, et al.2018(20) | 63/F | Japan | melanoma | nivolumab | 8 | + | 0.17 | 8.6 | NA | (+) |
| 23 | Magis, et al.2018(21) | 41/F | France | melanoma | pembrolizumab | 19 | + | 0.03 | 6.8 | GADA (-), IA-2A (+), IAA (-) | (+) |
| 24 | Magis, et al.2018(21) | 48/M | France | melanoma | nivolumab | 22 | + | 0.08 | 6.4 | GADA (-), IA-2A (-) | (+) |
| 25 | Magis, et al.2018(21) | 48/F | France | melanoma | nivolumab | 42 | + | 0.25 | 7.6 | GADA(-), IA-2A (+)，IAA (-) | (+) |
| 26 | Magis, et al.2018(21) | 22/M | France | melanoma | nivolumabp or ipilimumab | 14 | + | 0.45 | 7.08 | GADA (-), IA-2A (-) | (-) |
| 27 | Atkins, et al.2018(22) | 50/M | Canada | tonsillar carcinoma | avelumab | 1 | + | 0.189 | 6.4 | GADA (+), IAA (-) | N/A |
| 28 | Cheng S, et al.2018(23) | 53/M | China | esophageal carcinoma | N/A | 5 | + | 0.03 | 8.3 | ICA (-), IAA (-), GADA (-) | N/A |
| 29 | Sum, M, et al.2018 (24) | 75/M | USA | melanoma | ipilimumab and nivolumab | 3 | - | 0.3 | 7.6 | GADA (+), IAA (-), ICA(-) | N/A |
| 30 | Gunawan, et al.2018 (25) | 52/M | Australia | melanoma | ipilimumab and nivolumab | 3 | + | 0.15 | 7.7 | GADA (-), IA-2A (-), ZnT8 (-) | N/A |
| 31 | Omodaka, et al.2018(26) | 64/F | Japan | melanoma | ipilimumab and nivolumab | 4 | - | 0.02 | 6.7 | GADA (-), IA-2A (-) | N/A |
| 32 | Clotman K, et al.2018(27) | 73/F | Belgium | melanoma | pembrolizumab | 2 | + | 0.3 | 7.1 | GADA (+), ICA(+), IA-2A (-), ZnT8 (-) | (+) |
| 33 | Venetsanaki，et al.2019(28) | 71/M | Greece | lung cancer | nivolumab | N/A | - | 0.1 | 7.8 | ICA (-), GADA (-) | (-) |
| 34 | Okahata, et al.2019(29) | 52/F | Japan | breast cancer | nivolumab | 10 | - | 0.3 | 7.8 | IAA (-), GADA (-), IA-2A (-) | (-) |
| 35 | Yamamoto, et al.2019(30) | 77/M | Japan | renal cell carcinoma | nivolumab | 6 | - | 0.3 | 6.2 | IAA (-), GADA (-), IA-2A (-), ZnT8 (-) | (+) |
| 36 | Saito, et al.2019(31) | 82/M | Japan | lung cancer | pembrolizumab | 16 | - | 0.01 | 6.1 | (-) | (-) |
| 37 | Shibayama et al.2019(32) | 81/F | Japan | merkel cell carcinoma | avelumab | 10 | - | 0.08 | 7.5 | GADA (-), IA-2A (-) | (+) |
| 38 | Tohi et al.2019(33) | 75/M | Japan | urothelial carcinoma | pembrolizumab | 3 | - | 0.01 | 6.7 | GADA (-) | N/A |
| 39 | Edahiro, et al.2019(34) | 61/F | Japan | lung cancer | pembrolizumab | 8 | + | 0.1 | 8.4 | GADA (-) | N/A |
| 40 | Tassone, et al.2019(35) | 42/M | Italy | lung cancer | nivolumab | 4 | + | 0.2 | 6.0 | GADA(+),ICA(-), IA-2A (-) | (+) |
| 41 | Marchand, et al.2019(36) | 55/M | France | lung cancer | nivolumab | 9 | + | 0.3 | 8.2 | GADA (-),  IA-2A (-), ZnT8 (-) | (-) |
| 42 | Marchand, et al.2019(36) | 69/M | France | lung cancer | durvalumab | 13 | + | 0.3 | 7.4 | GADA (-), IA-2A (-), ZnT8 (-) | (-) |
| 43 | Marchand, et al.2019(36) | 65/M | France | melanoma | pembrolizumab | 12 | + | 0.3 | 8.5 | GADA(-),IA-2A(-),ZnT8(-) | (-) |
| 44 | Marchand, et al.2019(36) | 65/F | France | melanoma | nivolumab | 5 | + | 0.3 | 7.3 | GADA (-), IA-2A (-), ZnT8 (-) | (-) |
| 45 | Skorpen et al.2019(37) | 65/M | Norway | lung cancer | pembrolizumab | 2 | + | 0.02 | 8.4 | GADA(-),IA-2A(-), IAA (-) | N/A |
| 46 | Kotwal et al.2019(38) | (-)/(-) | USA | N/A | pembrolizumab | 1 | + | 0.1 | 7.8 | GADA(+),IA-2A(+), IAA (+), ZnT8 (-) | N/A |
| 47 | Maamari J et al.2019(39) | 47/F | USA | cardiac angiosarcoma | pembrolizumab. | 1 | + | 0.1 | 6.4 | GADA (+), ICA (-) | N/A |
| 48 | Hakami et al.2019(40) | 52/M | Ireland | melanoma | pembrolizumab | 7 | + | 0.01 | 8.3 | GADA (-), ICA (-) | N/A |
| 49 | Mengíbar et al.2019(41) | 55/M | Spain | urothelial carcinoma | durvalumab | 1 | + | 0.02 | 8.4 | GADA(+),IA-2A (+) | N/A |
| 50 | Hailuan Z.2019(42) | 52/M | China | renal cancer | nivolumab | 6 | + | 0.01 | 8.2 | ICA (-), IAA (-), GADA (-) | N/A |
| 51 | Miyauchi, et al.2020(43) | 79/M | Japan | lung cancer | nivolumab | 1 | - | 0.01 | 6.1 | GADA (-) | (+) |
| 52 | Kusuki et al.2020(44) | 72/M | Japan | lung cancer | pembrolizumab | 5 | + | 0.03 | 8.1 | GADA (-) | (+) |
| 53 | Gu Z, et al,2020(45) | 52/M | China | lung cancer | N/A | 18 | + | 0.01 | 8.1 | ICA (-), IAA (-), GADA (-)，IA-2A (-) | (+) |
| 54 | Zhang R, et al,2020(46) | 28/F | China | osteosarcoma | pembrolizumab | 5 | + | 0.01 | 6.1 | IAA (+), GADA (-), ICA (-) | N/A |
| 55 | Singh V,et al,2020(47) | 65/F | USA | renal carcinoma | ipilimumab and nivolumab | 4 | + | 0.1 | 8.5 | GADA (-), IA-2A (-), ZnT8 (-), IAA (-) | N/A |
| 56 | Shi Y,et al,  2020(48) | 54/M | China | colon cancer | toripalimab | 5 | + | 0.06 | 8.0 | GADA (-), IA-2A (-),IAA (-), ICA (-) | N/A |
| 57 | Wen L, et al,2020(49) | 56/M | China | liver Cancer | sintilimab | 8 | + | 0.21 | 7.8 | ICA (-), IAA (-), GADA (-) | (-) |
| 58 | He T,et al,2020(50) | 55/M | China | liver Cancer | N/A | N/A | - | 0.01 | 7.6 | (-) | N/A |
| 59 | Hong AR, et al,2020(51) | 65/F | Korea | biliary cancer | pembrolizumab | 7 | + | 0.1 | 5.8 | IAA (-), GADA (-) | N/A |
| 60 | Nishioki T,et al,2020(52) | 73/F | Japan | lung cancer | atezolizumab | 2 | + | urinary C-peptide0.7ug/d | 7.3 | GADA (-), IA-2A (-) | N/A |
| 61 | Kyriacou, A., et al，2020(53) | 68/F | UK  (South-East Asian origin ) | lung cancer | pembrolizumab | N/A | + | 0.1 | 7.0 | GADA (-), IA-2A (-), ZnT8 (-) | N/A |
| 62 | Keerty, D , et al， 2020(54) | 49/F | USA | lung cancer | nivolumab and ipilimumab | N/A | + | 0.1 | 6.6 | GADA (-), IAA (-) | N/A |
| 63 | Baroud, S, et al.2020(55) | 67/F | USA | melanoma | nivolumab | N/A | + | 0.05 | 7.0 | GADA (+) | N/A |
| 64 | Kedzior SK, et al.2021(56) | 51/F | USA | lung cancer | pembrolizumab | 2 | + | 0.1 | 8.3 | GADA (+) | N/A |
| 65 | Boswell L,et al,2021(57) | 51/M | Spain | melanoma | nivolumab and ipilimumab | 13 | - | 0.02 | 7.9 | GADA (-), IA-2A (-),IAA (-) | (-) |
| 66 | Yaura K, et al.2021(58) | 60/F | Japan | renal carcinoma | nivolumab and ipilimumab | 3 | + | 0.25 | 6.5 | GADA (-) | (-) |
| 67 | Nikouline A, et al,2021(59) | 87/F | Canada | melanoma | nivolumab | 3 | + | 0.01 | 8.5 | N/A | N/A |
| 68 | Ishi A,et al,2021(60) | 62/M | Japan | lung cancer | atezolizumab | 1 | + | 0.2 | 6.9 | GADA (-), IAA (-) | (+) |
| 69 | Huang X, et al,2021(61) | 59/M | China | lung cancer | sintilimab | 5 | + | 0.01 | 7.4 | GADA(-), IAA (-) | N/A |
| 70 | Tang Y, et al,2021(62) | 61/F | China | breast cancer | pembrolizumab | 7 | + | 0.05 | 8.4 | ICA(-),IAA(-),GADA(-),  IA-2A (-) | N/A |
| 71 | Chen YF.2021(63) | 63/M | China | lung cancer | sintilimab | 5 | + | 0.03 | 6.8 | ICA (-), IAA (-), GADA (-), IA-2A (-) | N/A |
| 72 | Xu GJ, et al, 2021(64) | 68/M | China | stomach cancer | camrelizumab | 6 | + | 0.03 | 8.0 | (-) | N/A |
| 73 | Shao YM, et al,2021(65) | 55/F | China | lung cancer | nivolumab | 6 | - | 0.3 | 7.1 | ICA (-), IAA (-), GADA (-) | N/A |
| 74 | Shao YM, et al,2021(65) | 55/M | China | esophageal carcinoma | sintilimab | 10 | + | 0.01 | 7.7 | IAA (-) | N/A |
| 75 | Shao YM, et al,2021(65) | 64/F | China | lung cancer | nivolumab | 15 | + | 0.03 | 8.1 | (-) | N/A |
| 76 | Qian GF, et al,2021(66) | 65/M | China | lung cancer | sintilimab | 4 | + | 0.01 | 6.8 | ICA (-), IAA (-), GADA (-) | (-) |
| 77 | Liu XY, et al,2021(67) | 70/F | China | stomach cancer | camrelizumab | N/A | + | 0.04 | 7.88 | IA-2A (+) | N/A |
| 78 | Our case | 52/M | China | lung cancer | sintilimab | 8 | + | 0.01 | 7.8 | GADA(-),IA-2A(-),ZnT8(-) | N/A |
| 79 | Our case | 66/M | China | lung cancer | sintilimab | 4 | + | 0.01 | 8.2 | NA | N/A |
| 80 | Our case | 64/F | China | renal cancer | pembrolizumab | 5 | + | 0.01 | 7.24 | GADA(-),IA-2A(-),ZnT8(-) | N/A |

Islet antibodies, including Islet cell autoantibodies (ICAs), Glutamic acid decarboxylase autoantibody (GADA), Protein tyrosine phosphatase autoantibody (IA-2A)

Zinc transporter 8 autoantibody (ZnT8A)

DKA diabetic ketoacidosis

N/A not applicable

**Supplemental table 2**  Summary of Case Reports of Immune checkpoint inhibitor induced type 1 diabetes (IT1D)

| case | Literature | Onset Age/sex | country | Primary  diagnosis | ICI drugs | Doses | DKA | Diagnosis | | Islet antibodies | HLA susceptibility genes for type 1 diabetes |
| --- | --- | --- | --- | --- | --- | --- | --- | --- | --- | --- | --- |
|  |  |  |  |  |  |  |  | C-peptide  (ng/ml) | HbA1c  (%) |  |  |
| 1 | Hughes, et al. 2015(1) | 63/M | USA | renal cancer | nivolumab | N/A | + | 1.3 | 8.2 | GADA (+), ICA (+), IAA (+) | (+) |
| 2 | Hughes, et al. 2015(1) | 64/F | USA | melanoma | pembrolizumab | N/A | - | 0.5 | 7.4 | ICA (-), IAA (-), GADA (-) | (+) |
| 3 | MellatiM, et al.2015(68) | 70/M | USA | lung cancer | Anti-PD-L1 | 5 | + | 0.3 | 9.8 | ICA (-), GADA (-) | N/A |
| 4 | Mellati, M. et al.2015(68) | 66/F | USA | jaw cancer | Anti-PD-L1 | 3 | N/A | 0.1 | 9.4 | GADA (+), IAA (-), ICA(-), ZnT8 (-) | (+) |
| 5 | Martin-Liberal J, et al.2015(69) | 54/F | UK | melanoma | pembrolizumab | 3 | + | N/A | N/A | GADA (+), ICA(-) | (+) |
| 6 | Hansen E,et al.2016(70) | 58/M | USA | melanoma | pembrolizumab | 17 | N/A | 2.4 | 9.7 | GADA (+), ICA(-) | N/A |
| 7 | Ikegami H, et al.(71) | 70/M | Japan | N/A | nivolumab | N/A | - | 0.02 | 9.2 | GADA (-), IA-2A (-), ZnT8 (-) | N/A |
| 8 | Chae, Y. K. et al.2017(16) | 76/M | USA | lung cancer | pembrolizumab | 1 | - | 0.81 | 5.8 | GADA (+), IA-2A (+),IAA (-) | N/A |
| 9 | Teramoto, Y. et al.2017(72) | 63/F | Japan | melanoma | nivolumab | 8 | + | 0.08 | 8.9 | IAA (-), GADA (-), IA-2A (-), | N/A |
| 10 | Gauci ML, et al. 2017(73) | 73/M | France | melanoma | nivolumab | 3 | N/A | 0.1 | 8.8 | IAA(+),GADA(+),IA-2A (+) | N/A |
| 11 | Kumagai R et al.2017(74) | 73/M | Japan | lung cancer | nivolumab | N/A | - | 0.97 | 9.4 | GADA (-), IA-2A (-),ICA (-), IAA (-), ZnT8 (-) | (+) |
| 12 | Hickmott L, et al.2017(75) | 57/M | UK | urothelial carcinoma | atezolizumab | 5 | + | 0.65 | 7.5 | ICA (-), IAA (-), GADA (-) | (+) |
| 13 | Way J,et al,2017(76) | 84/F | USA | nasopharyngeal carcinoma | durvalumab | 11 | - | 0.4 | 9.1 | GADA (+) | N/A |
| 14 | Way J,et al,2017(76) | (N/A)/M | USA | prostate cancer | atezolizumab | N/A | - | 1.1 | 8.2 | GADA (+), ICA (+) | N/A |
| 15 | Magis et al.2018(21) | 50/M | France | melanoma | nivolumab | N/A | + | 0.63 | 7.51 | GADA (-), IA-2A (-) | (+) |
| 16 | Villarreal J, et al,2018(77) | 66/M | USA | melanoma | nivolumab and ipllimumab | 1 | - | 0.6 | N/A | GADA(+), IA-2A(-) | N/A |
| 17 | Capitao, R.et al.2018(78) | 74/F | Portugal | lung cancer | nivolumab | 2 | + | 0.2 | 8.7 | GADA (+) | N/A |
| 18 | Gunjur A, et al.2019(79) | 78/F | Australia | melanoma | pembrolizumab | 1 | + | 0.66 | 6.9 | GADA (+), IA-2A (+) | (+) |
| 19 | Boyle V, et al,2019(80) | 56/M | New Zealand | melanoma | pembrolizumab | N/A | N/A | 2.15 | 7.4 | GADA (-), IA-2A (-) | (-) |
| 20 | Marchand L, et al.2019(36) | 72/M | France | lymphoma | nivolumab | 3 | - | 3 | 11.4 | GADA (-), IA-2A (+), ZnT8 (-) | (-) |
| 21 | Hatakeyama Y et al.2019(81) | 60/M | Japan | lung cancer | nivolumab | 36 | - | 0.78 | 9.1 | GADA (-), IA-2A (-) | N/A |
| 22 | Mancano MA et al.2019(82) | 61/F | USA | cholangiocarcinoma | pembrolizumab | 6 | + | N/A | 8.7 | GADA (+) | N/A |
| 23 | Kotwal et al.2019(38) | N/A | USA | N/A | pembrolizumab | 2 | + | 0.9 | 8.6 | GADA(+),IA-2A(-),IAA (-) | N/A |
| 24 | Kotwal et al.2019(38) | N/A | USA | N/A | pembrolizumab | 1 | + | 0.1 | 10.7 | GADA(+),IA-2A(-),IAA (-) | N/A |
| 25 | Kotwal et al.2019(38) | N/A | USA | N/A | pembrolizumab | 3 | - | 0.3 | 10.5 | GADA (-),IA-2A (-), IAA (+), ZnT8 (-) | N/A |
| 26 | Kotwal et al.2019(38) | N/A | USA | N/A | pembrolizumab | 6 | N/A | 5.2 | 9.7 | N/A | N/A |
| 27 | Kotwal et al.2019(38) | N/A | USA | N/A | pembrolizumab | 5 | + | N/A | 9.7 | GADA (-) | N/A |
| 28 | Kotwal et al.2019(38) | N/A | USA | N/A | pembrolizumab | 17 | + | 0.7 | 7.8 | GADA (+), IA-2A (-), IAA (-), ZnT8 (-) | N/A |
| 29 | Kotwal et al.2019(38) | N/A | USA | N/A | pembrolizumab | 16 | + | N/A | 11.3 | N/A | N/A |
| 30 | Kotwal et al.2019(38) | N/A | USA | N/A | pembrolizumab | 4 | + | N/A | 11.2 | N/A | N/A |
| 31 | Kotwal et al.2019(38) | N/A | USA | N/A | pembrolizumab | 2 | + | 0.4 | 8.8 | N/A | N/A |
| 32 | Kotwal et al.2019(38) | N/A | USA | N/A | pembrolizumab | 4 | - | N/A | 10.6 | GADA (-), IA-2A (-), IAA (-), ZnT8 (-) | N/A |
| 33 | Marchand et al.2019(36) | 83/M | France | melanoma | pembrolizumab | 4 | - | 3 | 9.4 | GADA (-), IA-2A (-), ZnT8 (-) | (+) |
| 34 | Sakaguchi, C.et al.2019(83) | 68/F | Japan | melanoma | nivolumab | 28 | + | 0.2 | 8.2 | GADA (-), IA-2A (-),ICA (-), IAA (-), ZnT8 (-) | (+) |
| 35 | Lanzolla G, et al.2019(84) | 60/M | Italy | lung cancer | atezolizumab | 4 | + | 0.7 | N/A | GADA (-), IA-2A (-) | N/A |
| 36 | Yilmaz M. 2020(85) | 49/M | Turkey | renal cancer | nivolumab | 21 | + | 0.24 | 10.9 | IAA (-), GADA (-), ICA (-), | N/A |
| 37 | Zhang R et al,2020(46) | 59/F | China | lung cancer | nivolumab | 10 | + | 0.72 | 9.8 | IAA (-), GADA (-), ICA (-), | N/A |
| 38 | Kurihara S, et al.2020(86) | 48/M | Japan | parotid cancer | nivolumab | 6 | - | 1.55 | 7.2 | GADA (-), IA-2A (-), ZnT8 (-) | (+) |
| 39 | Samoa RA, et al.2020(87) | 12/M | Spain | Hodgkin’s lymphoma | pembrolizumab | 5 | + | 0.3 | 8.9 | GADA(-),IA-2A(+),IAA (+) | (+) |
| 40 | Yun K, et al.2020(88) | 67/M | USA | lung cancer | nivolumab | N/A | + | N/A | 9.2 | GADA (-), ICA (-),IAA (-) | N/A |
| 41 | Yun K, et al.2020(88) | 49/M | USA | lung cancer | nivolumab | N/A | + | N/A | 8.9 | GADA (+) | N/A |
| 42 | Yun K, et al.2020(88) | 74/F | USA | lung cancer | nivolumab | N/A | + | N/A | 9.3 | GADA (-), ICA (-), IAA (-) | N/A |
| 43 | Hong AR, et al.2020(51) | 76/M | Korea | lung cancer | pembrolizumab | 11 | + | 0.01 | 10.4 | GADA (-), IAA (-) | N/A |
| 44 | Hong AR, et al.2020(51) | 67/M | Korea | urothelial carcinoma | atezolizumab | 9 | + | 0.01 | 9.8 | GADA (-) | N/A |
| 45 | Marshall S, et al.2020(89) | 70/M | Japan | stomach cancer | nivolumab | 8 | - | 0.03 | 9.4 | GADA (-), IA-2A (-) | N/A |
| 46 | Hughes MS, et al.2020(90) | 48/M | USA | Hodgkin’s lymphoma | nivolumab | N/A | N/A | 1 | 7.1 | GADA (+),IA-2A (-),IAA (-) | (+) |
| 47 | Mae S, et al. 2021(91) | 59/M | Japan | stomach cancer | nivolumab | 12 | + | N/A | 10.6 | GADA (-), IA-2A (-),IAA (-),  ZnT8 (-) | (-) |
| 48 | Kethireddy N, et al.2021(92) | 88/M | USA | melanoma | pembrolizumab | 3 | - | N/A | 9.0 | GADA (+) | N/A |
| 49 | Tan YJ et al.2021(93) | 68/M | China | lung cancer | sintilimab | 12 | - | 0.312 | 8.8 | GADA (-), ICA (+), IAA (-) | N/A |
| 50 | Dou WJ, et al.2021(94) | 72/M | China | stomach cancer | carrellizumab | 4 | - | 0.1 | 9.4 | (-) | N/A |
| 51 | Yang F,et al.2021(95) | 47/M | China | melanoma | toripalimab | 5 | - | 0.54 | 8.0 | (-) | N/A |
| 52 | You LN, et al.2021(96) | 58/M | China | lung cancer | durvalumab | 7 | - | 1.08 | 7.54 | (-) | N/A |
| 53 | Jiang L, et al.2021(97) | 75/M | China | lung cancer | pembrolizumab | 5 | + | 0.434 | 9.72 | ICA (-), IAA (-), GADA (-) | N/A |
| 54 | Kikuchi F, et al.2021(98) | 62/M | Japan | renal cancer | nivolumab | 10 | - | 0.2 | 8.1 | GADA (-) | N/A |
| 55 | Takata M, et al,2021(99) | 59/M | Japan | melanoma | nivolumab | 18 | - | 1.69 | 7.5 | N/A | N/A |
| 56 | Our case | 46/M | China | nasopharyngeal carcinoma | toripalimab | 13 | + | 0.46 | 12.6 | GADA (+), IA-2A (-),ICA (-),  ZnT8 (-) | N/A |

Islet antibodies, including Islet cell autoantibodies (ICAs), Glutamic acid decarboxylase autoantibody (GADA), Protein tyrosine phosphatase autoantibody (IA-2A)

Zinc transporter 8 autoantibody (ZnT8A)

DKA diabetic ketoacidosis

N/A not applicable

**Supplemental table 3**  Logistic regression analysis

| Variable | B value | SE | P | OR(95%CI) |
| --- | --- | --- | --- | --- |
| Ethnic origin | 1.437 | 0.854 | 0.092 | 4.209(0.789, 22.44) |
| Islet autoantibodies | 0.217 | 0.859 | 0.8 | 1.243(0.231, 6.696) |
| Susceptibility genes | -1.458 | 0.875 | 0.095 | 0.233(0.042, 1.292) |

**References**

1. Hughes J, Vudattu N, Sznol M, Gettinger S, Kluger H, Lupsa B, et al. Precipitation of autoimmune diabetes with anti-PD-1 immunotherapy. Diabetes Care. 2015;38(4):e55-7.DOI: 10.2337/dc14-2349

2. Gaudy C, Clévy C, Monestier S, Dubois N, Préau Y, Mallet S, et al. Anti-PD1 Pembrolizumab Can Induce Exceptional Fulminant Type 1 Diabetes. Diabetes Care. 2015;38(11):e182-3.DOI: 10.2337/dc15-1331

3. Miyoshi Y, Ogawa O, Oyama Y. Nivolumab, an Anti-Programmed Cell Death-1 Antibody, Induces Fulminant Type 1 Diabetes. Tohoku J Exp Med. 2016;239(2):155-8.10.DOI: 1620/tjem.239.155

4. Aleksova J, Lau PK, Soldatos G, McArthur G. Glucocorticoids did not reverse type 1 diabetes mellitus secondary to pembrolizumab in a patient with metastatic melanoma. BMJ Case Rep. 2016;2016.DOI: 10.1136/bcr-2016-217454

5. Usui Y, Udagawa H, Matsumoto S, Imai K, Ohashi K, Ishibashi M, et al. Association of Serum Anti-GAD Antibody and HLA Haplotypes with Type 1 Diabetes Mellitus Triggered by Nivolumab in Patients with Non-Small Cell Lung Cancer. J Thorac Oncol. 2017;12(5):e41-e43.DOI: 10.1016/j.jtho.2016.12.015

6. Munakata W, Ohashi K, Yamauchi N, Tobinai K. Fulminant type I diabetes mellitus associated with nivolumab in a patient with relapsed classical Hodgkin lymphoma. Int J Hematol. 2017;105(3):383-6.DOI: 10.1007/s12185-016-2101-4

7. Kong SH, Lee SY, Yang YS, Kim TM, Kwak SH. Anti-programmed cell death 1 therapy triggering diabetic ketoacidosis and fulminant type 1 diabetes. Acta Diabetol. 2016;53(5):853-6.DOI: 10.1007/s00592-016-0872-y

8. Li S, Zhang Y, Sun Z, Hu J, Fang C. Anti-PD-1 pembrolizumab induced autoimmune diabetes in Chinese patient: A case report. Medicine (Baltimore). 2018;97(45):e12907.DOI: 10.1097/MD.0000000000012907

9. Araújo M, Ligeiro D, Costa L, Marques F, Trindade H, Correia JM, et al. A case of fulminant Type 1 diabetes following anti-PD1 immunotherapy in a genetically susceptible patient. Immunotherapy. 2017;9(7):531-5.DOI: 10.2217/imt-2017-0020

10. Tsiogka A, Jansky GL, Bauer JW, Koelblinger P. Fulminant type 1 diabetes after adjuvant ipilimumab therapy in cutaneous melanoma. Melanoma Res. 2017;27(5):524-5.DOI: 10.1097/CMR.0000000000000384

11. Takahashi A, Tsutsumida A, Namikawa K, Yamazaki N. Fulminant type 1 diabetes associated with nivolumab in a patient with metastatic melanoma. Melanoma Res. 2018;28(2):159-60.DOI: 10.1097/CMR.0000000000000418

12. Teló GH, Carvalhal GF, Cauduro CGS, Webber VS, Barrios CH, Fay AP. Fulminant type 1 diabetes caused by dual immune checkpoint blockade in metastatic renal cell carcinoma. Ann Oncol. 2017;28(1):191-2.DOI: 10.1093/annonc/mdw447

13. Ishikawa K, Shono-Saito T, Yamate T, Kai Y, Sakai T, Shimizu F, et al. A case of fulminant type 1 diabetes mellitus, with a precipitous decrease in pancreatic volume, induced by nivolumab for malignant melanoma: analysis of HLA and CTLA-4 polymorphisms. Eur J Dermatol. 2017;27(2):184-5.DOI: 10.1684/ejd.2016.2923

14. Mizab Mellah C, Sánchez Pérez M, Santos Rey MD, Hernández García M. Fulminant type 1 diabetes mellitus associated with pembrolizumab. Endocrinol Diabetes Nutr. 2017;64(5):272-3.DOI: 10.1016/j.endinu.2017.01.005

15. Godwin JL, Jaggi S, Sirisena I, Sharda P, Rao AD, Mehra R, et al. Nivolumab-induced autoimmune diabetes mellitus presenting as diabetic ketoacidosis in a patient with metastatic lung cancer. J Immunother Cancer. 2017;5:40.DOI: 10.1186/s40425-017-0245-2

16. Chae YK, Chiec L, Mohindra N, Gentzler R, Patel J, Giles F. A case of pembrolizumab-induced type-1 diabetes mellitus and discussion of immune checkpoint inhibitor-induced type 1 diabetes. Cancer Immunol Immunother. 2017;66(1):25-32.DOI: 10.1007/s00262-016-1913-7

17. Leonardi GC, Oxnard GR, Haas A, Lang JP, Williams JS, Awad MM. Diabetic Ketoacidosis as an Immune-related Adverse Event from Pembrolizumab in Non-Small Cell Lung Cancer. J Immunother. 2017;40(6):249-51.DOI: 10.1097/CJI.0000000000000173

18. Sakurai K, Niitsuma S, Sato R, Takahashi K, Arihara Z. Painless Thyroiditis and Fulminant Type 1 Diabetes Mellitus in a Patient Treated with an Immune Checkpoint Inhibitor, Nivolumab. Tohoku J Exp Med. 2018;244(1):33-40.DOI: 10.1620/tjem.244.33

19. Shiba M, Inaba H, Ariyasu H, Kawai S, Inagaki Y, Matsuno S, et al. Fulminant Type 1 Diabetes Mellitus Accompanied by Positive Conversion of Anti-insulin Antibody after the Administration of Anti-CTLA-4 Antibody Following the Discontinuation of Anti-PD-1 Antibody. Intern Med. 2018;57(14):2029-34.DOI: 10.2169/internalmedicine.9518-17

20. Sakai G, Saito D, Nakajima R, Hatano M, Noguchi Y, Kurihara S, et al. Intrinsic insulin secretion capacity might be preserved by discontinuing anti-programmed cell death protein 1 antibody treatment in 'anti-programmed cell death protein 1 antibody-induced' fulminant type 1 diabetes. J Diabetes Investig. 2018;9(2):448-9.DOI: 10.1111/jdi.12662

21. Magis Q, Gaudy-Marqueste C, Basire A, Loundou A, Malissen N, Troin L, et al. Diabetes and Blood Glucose Disorders Under Anti-PD1. J Immunother. 2018;41(5):232-40.DOI: 10.1097/CJI.0000000000000218

22. Atkins PW, Thompson DM. Combination avelumab and utomilumab immunotherapy can induce diabetic ketoacidosis. Diabetes Metab. 2018;44(6):514-5.DOI: 10.1016/j.diabet.2017.05.005

23. Cheng S WZ, Lu Z, Shen L . Type 1 diabetes mellitus caused by anti-PD-1/PD-L1 therapy in a patient with esophageal squamous cell carcinoma: a case report and literature review. Journal of Multidisciplinary Cancer Management. 2018;4(1):69-76(in Chinese).

24. Sum M, Garcia FV. Immunotherapy-induced autoimmune diabetes and concomitant hypophysitis. Pituitary. 2018;21(5):556-7.DOI: 10.1007/s11102-018-0880-8

25. Gunawan F, George E, Roberts A. Combination immune checkpoint inhibitor therapy nivolumab and ipilimumab associated with multiple endocrinopathies. Endocrinol Diabetes Metab Case Rep. 2018;2018.DOI: 10.1530/EDM-17-0146

26. Omodaka T, Kiniwa Y, Sato Y, Suwa M, Sato M, Yamaguchi T, et al. Type 1 diabetes in a melanoma patient treated with ipilimumab after nivolumab. J Dermatol. 2018;45(10):e289-e90.DOI: 10.1111/1346-8138.14331

27. Clotman K, Janssens K, Specenier P, Weets I, De Block CEM. Programmed Cell Death-1 Inhibitor-Induced Type 1 Diabetes Mellitus. J Clin Endocrinol Metab. 2018;103(9):3144-54.DOI: 10.1210/jc.2018-00728

28. Venetsanaki V, Boutis A, Chrisoulidou A, Papakotoulas P. Diabetes mellitus secondary to treatment with immune checkpoint inhibitors. Curr Oncol. 2019;26(1):e111-e4.DOI: 10.3747/co.26.4151

29. Okahata S, Sakamoto K, Mitsumatsu T, Kondo Y, Noso S, Ikegami H, et al. Fulminant type 1 diabetes associated with Isolated ACTH deficiency induced by anti-programmed cell death 1 antibody-insight into the pathogenesis of autoimmune endocrinopathy. Endocr J. 2019;66(4):295-300.DOI: 10.1507/endocrj.EJ18-0328

30. Yamamoto N, Tsurutani Y, Katsuragawa S, Kubo H, Sunouchi T, Hirose R, et al. A Patient with Nivolumab-related Fulminant Type 1 Diabetes Mellitus whose Serum C-peptide Level Was Preserved at the Initial Detection of Hyperglycemia. Intern Med. 2019;58(19):2825-30.DOI: 10.2169/internalmedicine.2780-19

31. Saito D, Oikawa Y, Yano Y, Ikegami Y, Satomura A, Isshiki M, et al. Detailed Time Course of Decline in Serum C-Peptide Levels in Anti-Programmed Cell Death-1 Therapy-Induced Fulminant Type 1 Diabetes. Diabetes Care. 2019;42(3):e40-e1.DOI: 10.2337/dc18-1673

32. Shibayama Y, Kameda H, Ota S, Tsuchida K, Cho KY, Nakamura A, et al. Case of fulminant type 1 diabetes induced by the anti-programmed death-ligand 1 antibody, avelumab. J Diabetes Investig. 2019;10(5):1385-7.DOI: 10.1111/jdi.13022

33. Tohi Y, Fujimoto K, Suzuki R, Suzuki I, Kubota M, Kawakita M. Fulminant type 1 diabetes mellitus induced by pembrolizumab in a patient with urothelial carcinoma: A case report. Urol Case Rep. 2019;24:100849.DOI: 10.1016/j.eucr.2019.100849

34. Edahiro R, Ishijima M, Kurebe H, Nishida K, Uenami T, Kanazu M, et al. Continued administration of pembrolizumab for adenocarcinoma of the lung after the onset of fulminant type 1 diabetes mellitus as an immune-related adverse effect: A case report. Thorac Cancer. 2019;10(5):1276-9.DOI: 10.1111/1759-7714.13065

35. Tassone F, Colantonio I, Gamarra E, Gianotti L, Baffoni C, Magro G, et al. Nivolumab-induced fulminant type 1 diabetes (T1D): the first Italian case report with long follow-up and flash glucose monitoring. Acta Diabetol. 2019;56(4):489-90.DOI: 10.1007/s00592-018-1246-4

36. Marchand L, Thivolet A, Dalle S, Chikh K, Reffet S, Vouillarmet J, et al. Diabetes mellitus induced by PD-1 and PD-L1 inhibitors: description of pancreatic endocrine and exocrine phenotype. Acta Diabetol. 2019;56(4):441-8.DOI: 10.1007/s00592-018-1234-8

37. Skorpen PK, Margull J. Diabetic ketoacidosis following immunotherapy for lung cancer. Tidsskr Nor Laegeforen. 2019;139(4).DOI: 10.4045/tidsskr.18.0597

38. Kotwal A, Haddox C, Block M, Kudva YC. Immune checkpoint inhibitors: an emerging cause of insulin-dependent diabetes. BMJ Open Diabetes Res Care. 2019;7(1):e000591.DOI: 10.1136/bmjdrc-2018-000591

39. Maamari J, Yeung SJ, Chaftari PS. Diabetic ketoacidosis induced by a single dose of pembrolizumab. Am J Emerg Med. 2019;37(2):376.e1-.e2.DOI: 10.1016/j.ajem.2018.10.040

40. Hakami OA, Ioana J, Ahmad S, Tun TK, Sreenan S, McDermott JH. A case of pembrolizumab-induced severe DKA and hypothyroidism in a patient with metastatic melanoma. Endocrinol Diabetes Metab Case Rep. 2019;2019.DOI: 10.1530/EDM-18-0153

41. Mengíbar JL, Capel I, Bonfill T, Mazarico I, Espuña LC, Caixàs A, et al. Simultaneous onset of type 1 diabetes mellitus and silent thyroiditis under durvalumab treatment. Endocrinol Diabetes Metab Case Rep. 2019;2019(1).DOI: 10.1530/EDM-19-0045

42. Zeng H L, Gao X. One case of type 1 diabetes caused by PD-1 inhibitior and literature review. Chin J Endocrinol Metab. 2019;35(7):559-63(in Chinese).DOI: 10.3760/cma.j.issn.1000-6699.2019.07.004

43. Miyauchi M, Toyoda M, Zhang J, Hamada N, Yamawaki T, Tanaka J, et al. Nivolumab-induced fulminant type 1 diabetes with precipitous fall in C-peptide level. J Diabetes Investig. 2020;11(3):748-9.DOI: 10.1111/jdi.13143

44. Kusuki K, Suzuki S, Mizuno Y. Pembrolizumab-induced fulminant type 1 diabetes with C-peptide persistence at first referral. Endocrinol Diabetes Metab Case Rep. 2020;2020.DOI: 10.1530/EDM-19-0152

45. Gu Z, LW, Yang T, Liu X, Chen J. A case report of fulminant type1diabetes after treatment with programmed cell death protein‑1 antibody. Chin J Diabetes Mellitus. 2020;12(5):228-332(in Chinese).DOI: 10.3760/cma.j.cn115791-20191022-00382

46. Zhang R, LL, Cai X, Han X, Ji L. Two case reports of immune checkpoint inhibitors induced type1diabetes and literature review. Chinese Journal of Diabetes. 2020;28(1):62-70(in Chinese).DOI: 10.3969/j.issn.1006-6187.2020.01.013

47. Singh V, Chu Y, Gupta V, Zhao CW. A Tale of Immune-Related Adverse Events With Sequential Trials of Checkpoint Inhibitors in a Patient With Metastatic Renal Cell Carcinoma. Cureus. 2020;12(6):e8395.DOI: 10.7759/cureus.8395

48. Shi Y, SM, Liu KY, Yang T, Zhen XQ. One case of PD-1 inhibitor associated autoimmune polyendocrine syndrome and literature review. Chinese Journal of Endocrinology and Metabolism. 2020;36(12):1070-3(in Chinese).DOI:10.3760/cma.j.cn311282-20200225-00099

49. Wen L, Zou X, Chen Y, Bai X, Liang T. Sintilimab-Induced Autoimmune Diabetes in a Patient With the Anti-tumor Effect of Partial Regression. Front Immunol. 2020;11:2076.DOI: 10.3389/fimmu.2020.02076

50. He T, ZX, Fei YX, Gao L, Gong L, Zhang QL, Cheng GY. Anti-PD-1 therapy in advanced malignant liver tumor-induced type-1 diabetes mellitus: a case report[J]. Anti-PD-1 therapy in advanced malignant liver tumor-induced type-1 diabetes mellitus: a case report. Chinese Journal of Hepatology. 2020;28(6):518-20(in Chinese).DOI: 10.3760/ cma . j.cn 501113-20191124-00430

51. Hong AR, Yoon JH, Kim HK, Kang HC. Immune Checkpoint Inhibitor-Induced Diabetic Ketoacidosis: A Report of Four Cases and Literature Review. Front Endocrinol (Lausanne). 2020;11:14.DOI: 10.3389/fendo.2020.00014

52. Nishioki T, Kato M, Kataoka S, Miura K, Nagaoka T, Takahashi K. Atezolizumab-induced fulminant type 1 diabetes mellitus occurring four months after treatment cessation. Respirol Case Rep. 2020;8(9):e00685.DOI: 10.1002/rcr2.685

53. Kyriacou A, Melson E, Chen W, Kempegowda P. Is immune checkpoint inhibitor-associated diabetes the same as fulminant type 1 diabetes mellitus? Clin Med (Lond). 2020;20(4):417-23.DOI: 10.7861/clinmed.2020-0054

54. Keerty D, Das M, Hallanger-Johnson J, Haynes E. Diabetic Ketoacidosis: An Adverse Reaction to Immunotherapy. Cureus. 2020;12(9):e10632.DOI: 10.7759/cureus.10632

55. Baroud S, Mirza L. New-Onset Type 1 Diabetes Mellitus After Treatment With Nivolumab for Melanoma. Cureus. 2021;13(10):e18679. DOI: 10.7759/cureus.18679

56. Kedzior SK, Jacknin G, Hudler A, Mueller SW, Kiser TH. A Severe Case of Diabetic Ketoacidosis and New-Onset Type 1 Diabetes Mellitus Associated with Anti-Glutamic Acid Decarboxylase Antibodies Following Immunotherapy with Pembrolizumab. Am J Case Rep. 2021;22:e931702.DOI: 10.12659/AJCR.931702

57. Boswell L, Casals G, Blanco J, Jiménez A, Aya F, de Hollanda A, et al. Onset of fulminant type 1 diabetes mellitus following hypophysitis after discontinuation of combined immunotherapy. A case report. J Diabetes Investig. 2021;12(12):2263-6.DOI: 10.1111/jdi.13604

58. Yaura K, Sakurai K, Niitsuma S, Sato R, Takahashi K, Arihara Z. Fulminant Type 1 Diabetes Mellitus Developed about Half a Year after Discontinuation of Immune Checkpoint Inhibitor Combination Therapy with Nivolumab and Ipilimumab: A Case Report. Tohoku J Exp Med. 2021;254(4):253-6.DOI: 10.1620/tjem.254.253

59. Nikouline A, Brzozowski M. New DKA in a geriatric patient on immune checkpoint inhibitor therapy: a case report. Cjem. 2021;23(5):712-4.DOI: 10.1007/s43678-021-00145-4

60. Ishi A, Tanaka I, Iwama S, Sakakibara T, Mastui T, Kobayashi T, et al. Efficacies of programmed cell death 1 ligand 1 blockade in non-small cell lung cancer patients with acquired resistance to prior programmed cell death 1 inhibitor and development of diabetic ketoacidosis caused by two different etiologies: a retrospective case series. Endocr J. 2021;68(5):613-20.DOI: 10.1507/endocrj.EJ20-0769

61. Huang X, Yang M, Wang L, Li L, Zhong X. Sintilimab induced diabetic ketoacidosis in a patient with small cell lung cancer: A case report and literature review. Medicine (Baltimore). 2021;100(19):e25795.DOI: 10.1097/MD.0000000000025795

62. Tang Y, Zhao Z, Wang X, Zuo W, Zhang B, Yuan T, et al. A case of pembrolizumab-induced fulminant Type 1 diabetes mellitus in breast cancer. Immunotherapy. 2021;13(6):483-9.DOI: 10.2217/imt-2020-0222

63. YF C. Three cases of fulminant type 1 diabetes with ketoacidosis caused by immune checkpoint inhibitors. Journal of Practical Oncology. 2021;36(5):458-61(in Chinese).DOI:10.13267/j.cnki.syzlzz.2021.093

64. Xu GJ, ZQ, Chen Z. One case of diabetic ketoacidosis with thrombocytopenia caused by camrelizumab for injection. China Phannacist. 2021;24(3):531-4(in Chinese).DOI: 10.3969/j.issn.1008-049X.2021.03.025

65. Shao YM ZT, Gu N, Lu DF, Yuan GH. Immune-checkpoint inhibitors related diabetes: three cases report and literatures review. International Journal of Endocrinology and Metabolism. 2021;41(5):475-7(in Chinese).DOI:10. 3760/cma. j. cnl21383-2021623-06068

66. Qian Guofeng ZH, Zhou Weibin, Shen Jianguo. Fulminant type 1 diabetes induced by anti‑programmed cell death protein‑1 (PD‑1) antibody treatment: a case report. Chin J Intern Med. 2021;60(10):908-10(in Chinese).DOI: 10.3760/cma.j.cn112138-20210107-00017

67. Liu Xuyang LX, Zou Fang. One case of programmed death‑1 inhibitor‑induced fulminant type 1 diabetes and literature review. Chin J Diabetes Mellitus. 2021;13(10):1001-3(in Chinese).DOI: 10.3760/cma.j.cn115791-20210305-00130

68. Mellati M, Eaton KD, Brooks-Worrell BM, Hagopian WA, Martins R, Palmer JP, et al. Anti-PD-1 and Anti-PDL-1 Monoclonal Antibodies Causing Type 1 Diabetes. Diabetes Care. 2015;38(9):e137-8.DOI: 10.2337/dc15-0889

69. Martin-Liberal J, Furness AJ, Joshi K, Peggs KS, Quezada SA, Larkin J. Anti-programmed cell death-1 therapy and insulin-dependent diabetes: a case report. Cancer Immunol Immunother. 2015;64(6):765-7.DOI: 10.1007/s00262-015-1689-1

70. Hansen E, Sahasrabudhe D, Sievert L. A case report of insulin-dependent diabetes as immune-related toxicity of pembrolizumab: presentation, management and outcome. Cancer Immunol Immunother. 2016;65(6):765-7.DOI: 10.1007/s00262-016-1835-4

71. Ikegami H, Kawabata Y, Noso S. Immune checkpoint therapy and type 1 diabetes. Diabetol Int. 2016;7(3):221-7.DOI: 10.1007/s13340-016-0276-9

72. Teramoto Y, Nakamura Y, Asami Y, Imamura T, Takahira S, Nemoto M, et al. Case of type 1 diabetes associated with less-dose nivolumab therapy in a melanoma patient. J Dermatol. 2017;44(5):605-6.DOI: 10.1111/1346-8138.13486

73. Gauci ML, Laly P, Vidal-Trecan T, Baroudjian B, Gottlieb J, Madjlessi-Ezra N, et al. Autoimmune diabetes induced by PD-1 inhibitor-retrospective analysis and pathogenesis: a case report and literature review. Cancer Immunol Immunother. 2017;66(11):1399-410.DOI: 10.1007/s00262-017-2033-8

74. Kumagai R, Muramatsu A, Nakajima R, Fujii M, Kaino K, Katakura Y, et al. Acute-onset type 1 diabetes mellitus caused by nivolumab in a patient with advanced pulmonary adenocarcinoma. J Diabetes Investig. 2017;8(6):798-9.DOI: 10.1111/jdi.12627

75. Hickmott L, De La Peña H, Turner H, Ahmed F, Protheroe A, Grossman A, et al. Anti-PD-L1 atezolizumab-Induced Autoimmune Diabetes: a Case Report and Review of the Literature. Target Oncol. 2017;12(2):235-41.DOI: 10.1007/s11523-017-0480-y

76. Way J, Drakaki A, Drexler A, Freeby M. Anti-PD-L1 therapy and the onset of diabetes mellitus with positive pancreatic autoantibodies. BMJ Case Rep. 2017;2017.DOI: 10.1136/bcr-2017-220415

77. Villarreal J, Townes D, Vrablik M, Ro K. A Case of Drug-Induced Severe Endocrinopathies: What Providers in the Emergency Department Need to Know. Adv Emerg Nurs J. 2018;40(1):16-20.DOI: 10.1097/TME.0000000000000173

78. Capitao R, Bello C, Fonseca R, Saraiva C. New onset diabetes after nivolumab treatment. BMJ Case Rep. 2018;2018.DOI: 10.1136/bcr-2017-220999

79. Gunjur A, Klein O, Kee D, Cebon J. Anti-programmed cell death protein 1 (anti-PD1) immunotherapy induced autoimmune polyendocrine syndrome type II (APS-2): a case report and review of the literature. J Immunother Cancer. 2019;7(1):241.DOI: 10.1186/s40425-019-0713-y

80. Boyle V, Cundy T, Cutfield R. Rapid onset type 1 diabetes associated with the programmed cell death-1 inhibitor pembrolizumab. Intern Med J. 2019;49(7):930-1.DOI: 10.1111/imj.14340

81. Hatakeyama Y, Ohnishi H, Suda K, Okamura K, Shimada T, Yoshimura S. Nivolumab-induced acute-onset type 1 diabetes mellitus as an immune-related adverse event: A case report. J Oncol Pharm Pract. 2019;25(8):2023-6.DOI: 10.1177/1078155218816777

82. Mancano MA, Lapin J, Paik A. ISMP Adverse Drug Reactions: Pheochromocytoma Crisis Induced by Metoclopramide Baclofen Dependence Following High-Dose Therapy Fatal Cardiotoxicity Following High-Dose Cyclophosphamide Acute Anterograde Amnestic Syndrome Induced by Fentanyl Ivermectin-Induced Toxic Epidermal Necrolysis Pembrolizumab-Induced Type 1 Diabetes. Hosp Pharm. 2019;54(4):241-5.DOI: 10.1177/0018578719828860

83. Sakaguchi C, Ashida K, Yano S, Ohe K, Wada N, Hasuzawa N, et al. A case of nivolumab-induced acute-onset type 1 diabetes mellitus in melanoma. Curr Oncol. 2019;26(1):e115-e8.DOI: 10.3747/co.26.4130

84. Lanzolla G, Coppelli A, Cosottini M, Del Prato S, Marcocci C, Lupi I. Immune Checkpoint Blockade Anti-PD-L1 as a Trigger for Autoimmune Polyendocrine Syndrome. J Endocr Soc. 2019;3(2):496-503.DOI: 10.1210/js.2018-00366

85. Yilmaz M. Nivolumab-induced type 1 diabetes mellitus as an immune-related adverse event. J Oncol Pharm Pract. 2020;26(1):236-9.DOI: 10.1177/1078155219841116

86. Kurihara S, Oikawa Y, Nakajima R, Satomura A, Tanaka R, Kagamu H, et al. Simultaneous development of Graves' disease and type 1 diabetes during anti-programmed cell death-1 therapy: A case report. J Diabetes Investig. 2020;11(4):1006-9.DOI: 10.1111/jdi.13212

87. Samoa RA, Lee HS, Kil SH, Roep BO. Anti-PD-1 Therapy-Associated Type 1 Diabetes in a Pediatric Patient With Relapsed Classical Hodgkin Lymphoma. Diabetes Care. 2020;43(9):2293-5.DOI: 10.2337/dc20-0740

88. Yun K, Daniels G, Gold K, McCowen K, Patel SP. Rapid onset type 1 diabetes with anti-PD-1 directed therapy. Oncotarget. 2020;11(28):2740-6.DOI: 10.18632/oncotarget.27665

89. Marshall S, Kizuki A, Kitaoji T, Imada H, Kato H, Hosoda M, et al. Type 1 Diabetes, ACTH Deficiency, and Hypothyroidism Simultaneously Induced by Nivolumab Therapy in a Patient with Gastric Cancer: A Case Report. Case Rep Oncol. 2020;13(3):1185-90.DOI: 10.1159/000510044

90. Hughes MS, Pietropaolo M, Vasudevan MM, Marcelli M, Nguyen H. Checking the Checkpoint Inhibitors: A Case of Autoimmune Diabetes After PD-1 Inhibition in a Patient with HIV. J Endocr Soc. 2020;4(12):bvaa150.DOI: 10.1210/jendso/bvaa150

91. Mae S, Kuriyama A, Tachibana H. Diabetic Ketoacidosis as a Delayed Immune-Related Event after Discontinuation of Nivolumab. J Emerg Med. 2021;60(3):342-4.DOI: 10.1016/j.jemermed.2020.09.023

92. Kethireddy N, Thomas S, Bindal P, Shukla P, Hegde U. Multiple autoimmune side effects of immune checkpoint inhibitors in a patient with metastatic melanoma receiving pembrolizumab. J Oncol Pharm Pract. 2021;27(1):207-11.DOI: 10.1177/1078155220921543

93. Tan YJ FF, Liu F, Wang YB. Analysis of adverse reactions in a case of PD-1 inhibitor-induced polyendocrine syndrome. Central South Pharmacy. 2021;19(11):2470-2(in Chinese).DOI: 10.7539/j.issn.1672-2981.2021.11.042

94. Dou WJ LJ, Wang RF, Liang QQ, Du H. Drug analysis of a case of diabetic ketosis induced by anti-PD-1 treatment of cardiac cancer. Pharmaceutical and Clinical Research. 2021;29(2):140-2(in Chinese).

95. Yang F, ZX, Wang M, Yan YM. Clinical analysis of 2 cases of immune checkpoint inhibitor-related diabetes mellitus. Pharm care Res 2021;21(2):106-9(in Chinese).DOI: 10.5428/pcar20210206

96. You LN, WZ, Song LJ, Dai H, Ding WB, Zhu Z, Jiang K. One case of type 1 diabetes mellitus caused by durvalumab. Chin J New Drugs Clin Rem. 2021;40(11):792-4(in Chinese).DOI:10.14109/j.cnki.xyylc.2021.11.14

97. Jiang L HW. Diabetes mellitus induce by immune checkpoint inhibitors: a case report. Chin J Diabetes Mellitus. 2021;13(12):1174-6(in Chinese).DOI: 10.3760/cma.j.cn115791-20210511-00264

98. Kikuchi F, Saheki T, Imachi H, Kobayashi T, Fukunaga K, Ibata T, et al. Nivolumab-induced hypophysitis followed by acute-onset type 1 diabetes with renal cell carcinoma: a case report. J Med Case Rep. 2021;15(1):214.DOI: 10.1186/s13256-020-02656-7

99. Takata M, Nomura M, Yamamura K, Muto M, Komori T, Otsuka A, et al. Autoimmune polyendocrine syndrome type 3, characterized by autoimmune thyroid disease, type 1 diabetes mellitus, and isolated ACTH deficiency, developed during adjuvant nivolumab treatment. Asia Pac J Clin Oncol. 2021.DOI: 10.1111/ajco.13573
